# Effects of Sleep Deprivation on Working Memory: Change in Functional Connectivity Between the Dorsal Attention, Default Mode, and Fronto-Parietal Networks

**DOI:** 10.3389/fnhum.2020.00360

**Published:** 2020-10-12

**Authors:** Cimin Dai, Ying Zhang, Xiaoping Cai, Ziyi Peng, Liwei Zhang, Yongcong Shao, Cuifeng Wang

**Affiliations:** ^1^School of Psychology, Beijing Sport University, Beijing, China; ^2^The Eighth Medical Center of the General Hospital of People’s Liberation Army, Beijing, China; ^3^Department of Cadraword 3 Division, General Hospital of People’s Liberation Army, Beijing, China; ^4^Institute of Psychology, Chinese Academy of Sciences, Beijing, China; ^5^Suzhou Institute of Biomedical Engineering and Techology, Chinese Academy of Sciences, Suzhou, China; ^6^Department of Respiratory Medicine, Qingdao Huangdao People’s Hospital, Qingdao, China

**Keywords:** sleep deprivation, working memory, functional connectivity, brain network, fMRI

## Abstract

Sleep deprivation (SD) is very common in modern society and has a profound effect on cognitive function, in particular on working memory (WM). This type of memory is required for completion of many tasks and is adversely affected by SD. However, the cognitive neural mechanism by which SD affects WM, remains unclear. In this study, we investigated the changes in the brain network involved in WM after SD. Twenty-two healthy subjects underwent functional magnetic resonance imaging scan while in a state of resting wakefulness and again after 36 h of total SD and performed a WM task before each scanning session. Nineteen main nodes of the default mode network (DMN), dorsal attention network (DAN), fronto-parietal network (FPN), salience network (SN), and other networks were selected for functional analysis of brain network connections. Functional connectivity measures were computed between seed areas for region of interest (ROI)-to-ROI analysis and to identify patterns of ROI-to-ROI connectivity. The relationship between the significant changes in functional connectivity in the brain network and WM performance were then examined by Pearson’s correlation analysis. WM performance declined significantly after SD. Compared with the awake state, the functional connectivity between DAN and DMN significantly increased after SD while that between FPN and DMN significantly decreased. Correlation analysis showed that the enhanced functional connectivity between DAN and DMN was negatively correlated with the decline in WM performance and that the decline in functional connectivity between FPN and DMN was positively correlated with decreased WM performance. These findings suggested that SD may affect WM by altering the functional connectivity among DMN, DAN, and FPN.

## Introduction

Sleep is very important in both humans and animals. However, the pace and pressure of modern life is increasing, and an ever-increasing number of individuals are reporting sleep problems. Sleep deprivation (SD) has many negative physical and psychological effects, one of which is an adverse impact on cognitive function (Frenda et al., 2014; Boardman et al., 2018; Kusztor et al., 2019). Research on sleep disorders may help to improve our understanding of sleep and the negative outcomes associated with sleep disorders.

Working memory (WM) is a limited memory system that processes and temporarily stores information and plays an important role in many complex cognitive activities (Baddeley, 2010). It is also the basis for higher cognitive functions, including understanding, learning, and reasoning. The role of WM in advanced cognitive activities is important and much research effort has been focused on its composition and structure. Baddeley (2007) divided the WM system into three parts: the central executive system, which is the core component, and two other subordinate systems, the visuospatial sketchpad, which can maintain and operate visual and spatial information, and the phonological loop, which can passively store and actively retell voice information. Baddeley (2012) subsequently added another subordinate system, the episodic buffer, which can temporarily integrate multiple information from different coding systems. In addition to Baddeley’s multi-component WM model, there are other WM models, including Cowan’s embedded-processes model and Oberauer’s concentric model (Cowan, 1999; Oberauer, 2002; Chein et al., 2003). However, the most widely accepted model is the multi-component model devised by Baddeley.

The prefrontal cortex (PFC) is an important brain region for central executive function and is the core subcomponent of WM (Jones and Harrison, 2001; Jemery and Volker, 2019). A large number of studies have shown that the anterior cingulate cortex (ACC) and PFC, which includes the ventrolateral PFC (VLPFC) and the dorsolateral PFC (DLPFC), contain areas that are important for WM (Jansma et al., 2000; Collette and Linden, 2002; Curtis and D’esposito, 2003; Bunge et al., 2005; Badre and Wagner, 2007). For example, the DLPFC plays an important role in task coordination and attention distribution in executive control (Blumenfeld and Ranganath, 2007). Furthermore, there are many studies on the relationship between WM and functional connectivity in the brain (Luerding et al., 2008; Roberts et al., 2017). Many functional magnetic resonance imaging (fMRI) studies on the mechanism of WM in the brain suggest that resting-state fMRI is an effective tool for exploring the neural mechanisms of WM (Heuvel and Pol, 2010; Biswal, 2012).

Sleep deprivation causes a decline in many cognitive functions, one of the most sensitive of which is WM (Chee et al., 2006; Preece, 2012; Martínez-Cancino et al., 2015). Behavioral studies of WM after SD found that the longer the duration of SD, the longer the response time and the lower the accuracy rate (Lu, 2016; Tempesta et al., 2017; Harrington et al., 2018). fMRI studies have found that several brain regions, including the VLPFC, DLPFC, ACC, and posterior parietal cortex (PPC) are involved in processing of WM (Greicius et al., 2003; Owen et al., 2005; Badre and Wagner, 2007; Wu et al., 2014) and that the DLPFC, ACC, and posterior parietal lobe (PPL) are associated with WM load (Cappell et al., 2010; Yoon et al., 2016). There are changes in the connections between these brain regions after SD, as well as changes in the functional connectivity networks within these regions, which include the Default Mode Network, Dorsal Attention Network, Frontoparietal Network, and Salience Network (DMN, DAN, FPN, and SN, respectively) (Mu et al., 2005; Gujar et al., 2010; Havas et al., 2012). The changes in the functional connectivity in these brain regions may help us to understand the physiological basis of the effects of SD on WM. Therefore, these four brain function networks were our focus in this study.

Although there have many explorations of the neural mechanism involved in the decline in cognitive function and WM performance in sleep-deprived subjects, there is limited information on the changes in WM performance and the functional brain network after SD. The existing research has not clarified the impact of changes in network node functional connections. Analyses of WM based on a theoretical model should be closely related to the cognitive mechanism of attention but are rare. Therefore, this study selected the main nodes of the functional brain network that are closely related to WM to analyze the relationship between changes in the functional connections within these networks and the decrease in WM based on the WM model. Our research assumed that the mechanism of the decline in WM after SD was related to changes in functional connections between the FPN, DAN, and DMN.

## Materials and Methods

### Subjects

The study participants were recruited by advertisements placed at Beihang University. The inclusion criteria were right-handedness, normal or corrected vision, normal cognitive function, and at least average intelligence (confirmed by Combined Raven Test score of ≥50%). Participants were excluded if they had neurological or cardiovascular disease, cataract or glaucoma, pulmonary disease, hearing problems, as well as substance abusers. Participants also had to be free of sleep disorders. All participants were counseling to avoid smoking and drinking coffee and to aim for 8 h of regular sleep per day during the week before the experiment. Finally, 22 individuals were eligible to participate in the study. The study was approved by the ethics committee of The General Hospital of PLA (Beijing, China). All study participants fully understood the protocol and provided written informed consent before participating in the experiment.

### Experimental Paradigm

The experiment was performed at Beihang University. All study participants had access to medical staff for rescue intervention at any time outside the laboratory for the duration of the study. For continuous behavioral monitoring, we matched participants with a partner to help them stay awake at night. Participants could not leave the laboratory after the start of SD and were escorted to the scanning room.

**FIGURE 1:**
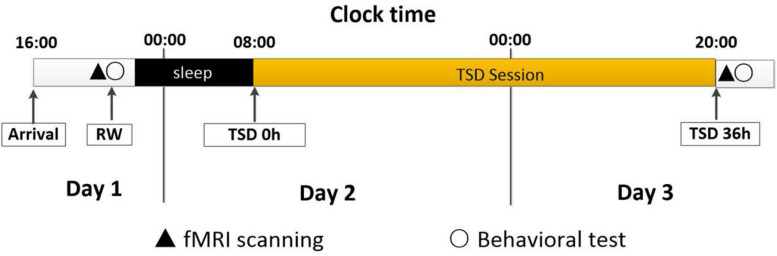
Experimental design and protocol. All the participants enter the laboratory at 16:00 on Day 1, and then complete the regular test. At approximately 20:00 on Day 1, fMRI scanning under RW condition will be done, followed by behavioral tests (working memory). After a routine nocturnal sleep period, TSD started at 8:00 on Day 2 and ended at 20:00 at on Day 3. During the whole TSD session, the participants needed to keep awaking for 36 h. The participants performed fMRI scanning after TSD at 20:00 on Day 3, and then had behavioral tests (working memory).

All the participants arrived at the laboratory at 4 pm on day 1 and completed a demographic questionnaire and the Self-Rating Anxiety Scale, Self-Rating Depression Scale, and Pittsburgh Sleep Quality Index tests. At approximately 8 pm on day 1, fMRI was performed when the subject was in a state of resting wakefulness (RW), after which WM tests were performed. After a routine nocturnal sleep period, total sleep deprivation (TSD) was started at 8 am on day 2 and ended at 8 pm on day 3. The study participants were required to remain awake for the entire 36-h TSD session (Terán-Pérez et al., 2012). The participants underwent fMRI scanning at 8 pm on day 3 and then repeated the WM tests. The experimental design is shown in Figure 1.

### WM Task Paradigm

In recent decades, the n-back paradigm has been widely used in the study of WM because the task is of moderate difficulty and sensitivity. This paradigm is often used to measure executive control in WM and to explore the neural mechanism of WM based on resting-state fMRI data (Martínez-Cancino et al., 2015; Soveri et al., 2017; Costers et al., 2020). The task requires the subject to monitor a stimulus sequence to determine if the stimulus currently presented is the same as that presented before several trials. So, participants need to timely monitor, update, and manipulate the information that is remembered (Seidman et al., 2006). Our study evaluated the WM performance of each subject through the 2-back task of visual WM. We adopted “Something Special” as the 2-back task with English letters as the external stimuli, although all the participants were native Chinese speakers. The participants received a series of visual letter stimuli on a computer screen and were asked to press the confirmation button on the keyboard as soon as possible, if the current letter was identical to that presented two trials back. The stimulus material included 15 upper case letters and 15 lower case letters (excluding letters similar to glyphs, such as L and M), a total of 122 letters with a letter size of 2.0 × 2.0 cm (width 1.5°, height 1.5°), and a test time of about 5 min. In the experiment, a prompt message (a white “+”) was displayed in the center of the display for a duration of 200 ms. After the prompt message had disappeared for 1 s, letters were displayed in the center of the screen one by one with a duration of 400 ms for each letter. The stimulus interval was 1600 ms. There were three 2-back blocks, each consisting of 30 stimuli, including 10 target letters. Before each task block started, an introduction instructed the subject how to perform the task. Finally, the study used the correct response per second to assess WM performance. The WM task was performed out of the scan and immediately after the fMRI scan was completed. To calculate the correct response per second, the most rapid 10% responses of each subject were selected as the most accurate test of WM, drawing lessons from the calculation method of the psychomotor vigilance test (PVT) indicator (Basner and Dinges, 2011; Chen et al., 2018).

### Resting State Paradigm

All participants underwent structural MRI and fMRI twice, in the resting state on both occasions. During the scans, participants were requested to stay awake with eyes open, remain still, and not think about anything in particular.

### Data Acquisition

Standard static functional images and T2^∗^ weighted echo-planar image (EPI) sequences were obtained at 309th Hospital of PLA using a 3.0T Signa scanner (Siemens AG, Munich, Germany) with a birdcage RF head coil. The scanning protocol consists of (i) localization, (ii) T1-flair anatomy (TR = 2 s, TE = 30 ms, flip angle = 12°, FoV = 256 × 256 mm, Sag slice number = 192, voxel size = 1 × 1 × 1 mm, no slice gap), and (iii) one resting-state session (TR = 2 s, TE = 30 ms, flip angle = 90°, FoV = 256 × 256 mm, matrix = 64 × 64, Slice = 35, Slice thickness = 3 mm, Slice gap = 1 mm). The scan duration was 480 s. The subject was placed in the scanner with the head comfortably restricted by foam padding to reduce head movement and earplugs to attenuate the noise of the scanner. During the resting-state scan, all participants was asked to keep their eyes open, lie as still as possible, and not think about anything too specific. A pulse oximeter was attached to a finger to record heart activity. All study participants also wore pressure bands around the waist to record breathing activity. The cardiac and respiratory signals collected were synchronized with the fMRI data to ensure that physiological changes would be removed during regression analysis. To ensure that participants did not fall asleep during the scan, the researcher monitored them using a camera and reminded them of the need to stay awake through a microphone before each scan if necessary. After each trial, the participants were asked if they had remained awake and all reported that they had done so.

### Data Preprocessing

The functional images were preprocessed using SPM 12 (University College London^1^) and CONN toolbox v17a (Neuroimaging Informatics Tools and Resources Clearinghouse^2^) software implemented in the 2016 version of MATLAB. CONN is a MATLAB-based cross-platform software for computing and analyzing functional connections in brain regions based on fMRI signals. This software has been widely used and its reliability has been well documented (Whitfield-Gabrieli and Nieto-Castanon, 2012). The first 10 volumes of the functional time series of each epoch were discarded so that the signal was stable, and participants were accustomed to scanning noise. Rotation and movement of all participants was within 2 mm or 2 degrees in the x, y, and z planes, respectively. Next, slice-timing and head-motion corrections were performed. Volumes were normalized to the standard EPI template in the Montreal Institute of Neuroscience space and restored to 3 × 3 × 3 mm. The resulting images were spatially smoothed with a 6 mm full width at half-maximum Gaussian kernel (Lu et al., 2020). In order to minimize the impact of motion and physiological noise factors, such as cardiac and respiratory signals, the CompCor approach was used for spatial and temporal preprocessing to define and remove confounds in the BOLD signal (Behzadi et al., 2007).

### Functional Connectivity Analysis

We scanned the ROI-ROI connectivity matrix, tested the hypotheses, and visualized data using the CONN toolbox implemented in the 2016 version of MATLAB version and SPM12. Nineteen ROIs from the DAN, DMN, FPN, and SN in the brain were drawn from the CONN software template (conn/rois/networks.nii). All the ROIs in the four networks were imported into the CONN toolbox. Functional connectivity measures were computed between seed areas for ROI-to-ROI analysis and to identify patterns of ROI-to-ROI connectivity. Partial correlation was used to estimate the functional connectivity between two nodes (Li et al., 2020). We compared functional connectivity between the RW and TSD scans using two-tailed paired *t*-tests. The significance of ROI-to-ROI connectivity was determined based on FDR-corrected *p*-Values. The analysis of TSD versus RW connectivity was set at an FDR-corrected *p*-Value of 0.05 for the voxel-level height threshold in both ROI-to-ROI and seed ROI tests.

### Behavioral Correlation

The relationship between the decline in WM and changes in functional connectivity were examined using correlation analysis. Correlations between changes in the correct response time (the 10% most rapid WM trials) and altered functional connectivity before and after SD were calculated. The significance level was kept at *p* < 0.05 to obtain the correlation between ROI and behavior.

## Results

### Physiological Data

Table 1 shows the demographic data, psychological features, and sleep characteristics of the study participants. All participants were monitored for breathing and heart rate during the fMRI scans. The paired *t*-test was used to compare the mean respiration and heart rate values in each subject before and after SD. No difference in the heart rate (*t* = −0.258, *p* > 0.05) or respiration rate (*t* = 0.960, *p* > 0.05) was found between the RW and TSD conditions (Table 1).

**TABLE 1:** Demographic data, psychological traits, and sleep evaluation.

	**RW state**	**TSD state**	***t***	***p***
Age (years)	24.00 ± 3.28	–	–	–
Male (n[%])	22 (100%)	–	–	–
BMI (kg/m^2^)	22.82 ± 1.98	–	–	–
Education (years)	16.14 ± 1.49	–	–	–
SAS	20.36 ± 10.81	–	–	–
SDS	22.82 ± 13.42	–	–	–
PSQI	3.36 ± 1.53			
Heart rate	71.09 ± 7.35	71.73 ± 7.86	-0.258	0.799
Respiratory rate	18.82 ± 2.28	18.18 ± 2.02	0.960	0.348
Correct response per second (WM)^a^	2.80 ± 0.36	2.47 ± 0.31	9.508	<0.0001

*t, paired *t*-test; BMI, Body mass index; SAS, Self-rating anxiety scale; SDS, Self-rating depression scale; PSQI, Pittsburgh sleep quality index; POMS, Profile of Mood States; WM, working memory; ^*a*^Correct response per second, the 10% fastest correct trials of working memory.*

### Changes in WM Performance Before and After SD

**FIGURE 2:**
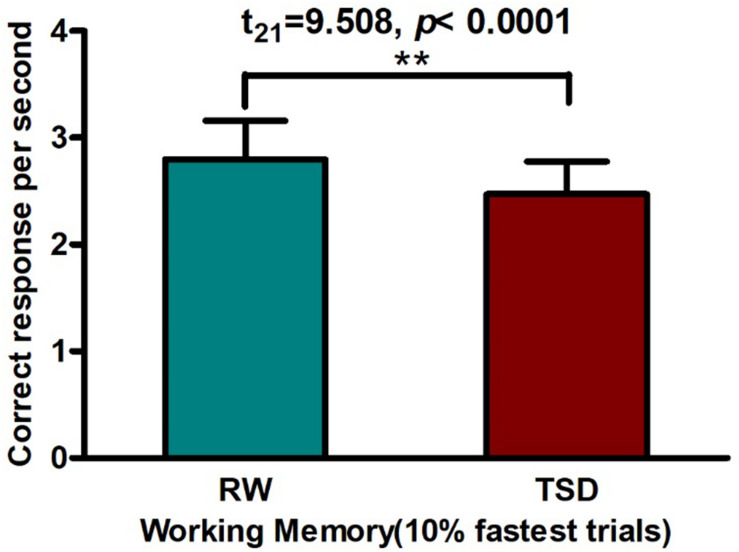
Worse working memory performance contrasting in RW vs. TSD: After 36 h TSD, correct response per second decreased by a paired *t*-test [**p < 0.0001]. TSD, total sleep deprivation; RW, rested wakefulness.

Using a paired *t*-test, we found that SD led to a significant decline in WM (*t*_21_ = 9.508, *p* < 0.0001, see Figure 2 and Table 1), which is consistent with previous studies (Drummond et al., 2012; Martínez-Cancino et al., 2015; Lu, 2016; Tempesta et al., 2017; Harrington et al., 2018).

### Resting Networks

**FIGURE 3:**
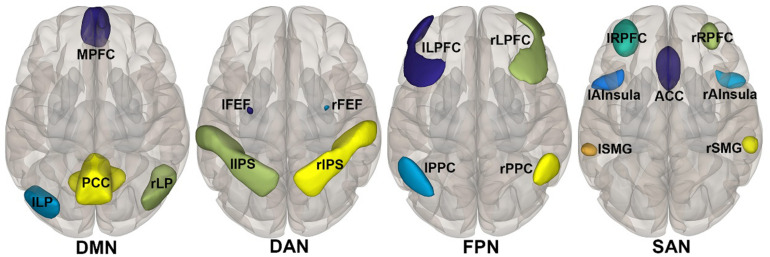
DMN, DAN, FPN, and SAN node location in resting Networks: According to the network template in CONN, 19 main nodes of the four networks related to working memory are selected, and their locations are shown in the figure. Their specific names and coordinates are shown in Table 2. DAN, dorsal attention network; DMN, default mode network; FPN, fronto-parietal network; SAN, salience network.

**FIGURE 4:**
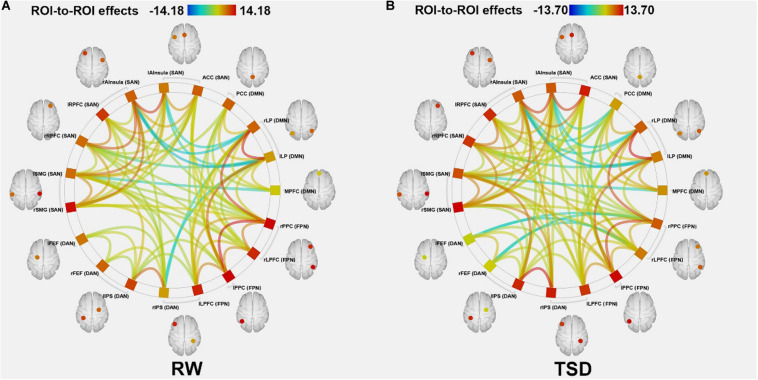
ROI-to-ROI connectivity of four networks in panels **(A)** RW and **(B)** TSD scans [*p* < 0.05 FDR set wise corrected for all comparisons across the entire network]. ROI, region of interest; TSD, total sleep deprivation; RW, rested wakefulness. DAN, dorsal attention network; DMN, default mode network; FPN, fronto-parietal network; SAN, salience network.

According to the CONN template, 19 brain regions in the DMN, DAN, FPN, and SN were selected as ROIs. The DMN includes the left and right paracentral lobules (lLP and rLP), medial prefrontal cortex (MPFC), and posterior cingulate cortex (PCC); the DAN includes the left and right frontal eye fields, left intraparietal sulcus, and right intraparietal sulcus (rIPS); the FPN includes the left lateral prefrontal cortex, right lateral prefrontal cortex (rLPFC), left and right posterior parietal cortices; and the SN includes the ACC, left and right anterior insulae, left and right rostrolateral prefrontal cortices, and left and right supramarginal gyri. Figure 3 shows the abbreviated names and locations of the 19 nodes. Their specific names and coordinates are shown in Table 2. The functional connections within and between the DAN, DMN, FPN and SAN in the RW and TSD states are shown in Figure 4 and Tables 3, 4.

**TABLE 2:** DMN, DAN, FPN and SN networks main node and location in resting Networks.

**network**	**ROI**	**ROI name**	**MNI center**
DMN	lLP	Left paracentral lobule	−39, −77,33
	MPFC	Medial prefrontal cortex	1,55, −3
	PCC	Posterior cingulate	1, −61,38
	rLP	Right paracentral lobule	47, −67,29
DAN	lFEF	Left frontal eye field	−27, −9,64
	lIPS	Left intraparietal sulcus	−39, −43,52
	rFEF	Right frontal eye field	30, −6,64
	rIPS	Right intraparietal sulcus	39, −42,54
FPN	lLPFC	Left lateral prefrontal cortex	−43,33,28
	lPPC	Left posterior parietal cortex	−46, −58,49
	rLPFC	Right lateral prefrontal cortex	41,38,30
	rPPC	Right posterior parietal cortex	52, −52,45
SN	ACC	Anterior cingulate	0,22,35
	lAInsula	Left anterior insula	−44,13,1
	lRPFC	Left rostrolateral prefrontal cortex	−32,45,27
	lSMG	Left supramarginal gyrus	−60, −39,31
	rAInsula	Right anterior insula	47,14,0
	rRPFC	Right rostrolateral prefrontal cortex	32,46,27
	rSMG	Right supramarginal gyrus	62, −35,32

*DAN, dorsal attention network; DMN, default mode network; FPN, fronto-parietal network; SN, salience network.*

**TABLE 3:** Resting state functional connectivity ROI pairs within- and between- DAN, DMN, FPN, and SN network for RW scans (*t*-test).

**Network**	**ROI-to-ROI**	***t***	**p-FDR**
Within-SN	ACC-lAInsula	7.3	0.0000
	ACC-lRPFC	3.89	0.0022
	ACC-lSMG	5.76	0.0001
	ACC-rAInsula	6.54	0.0000
	ACC-rRPFC	3.18	0.0089
	ACC-rSMG	2.92	0.0148
	lAInsula-lRPFC	6.02	0.0000
	lAInsula-lSMG	6.58	0.0000
	lAInsula-rAInsula	11.91	0.0000
	lAInsula-rRPFC	3.51	0.0053
	lAInsula-rSMG	5.09	0.0002
	lRPFC-lSMG	4.45	0.0013
	lRPFC-rAInsula	4.06	0.0025
	lRPFC-rRPFC	8.25	0.0000
	lRPFC-rSMG	3.85	0.0028
	lSMG-rAInsula	9.51	0.0000
	lSMG-rRPFC	3.77	0.0028
	lSMG-rSMG	7.87	0.0000
	rAInsula-rRPFC	10.58	0.0000
	rAInsula-rSMG	9.67	0.0000
	rRPFC-rSMG	6.96	0.0000
Within-FPN	lLPFC-lPPC	8.47	0.0000
	lLPFC-rLPFC	5.30	0.0001
	lLPFC-rPPC	6.79	0.0000
	lPPC-rLPFC	7.88	0.0000
	lPPC-rPPC	14.18	0.0000
	rLPFC-rPPC	8.58	0.0000
Within-DMN	lLP-MPFC	4.71	0.0003
	lLP-PCC	5.48	0.0001
	lLP-rLP	13.49	0.0000
	MPFC-PCC	3.30	0.0154
	MPFC-rLP	5.36	0.0005
	PCC-rLP	5.63	0.0002
Within-DAN	lFEF-lIPS	4.37	0.0048
	lFEF-rFEF	3.56	0.0168
	lFEF-rIPS	3.04	0.0371
	lIPS-rFEF	3.12	0.0187
	lIPS-rIPS	10.22	0.0000
	rFEF-rIPS	4.57	0.0030
SN-FPN	rPPC-rAInsula	2.36	0.0458
	rSMG-rLPFC	3.58	0.0040
	ACC-lPPC	4.54	0.0005
	lSMG-lPPC	3.73	0.0028
	lSMG-rLPFC	3.50	0.0043
	rSMG-rPPC	4.71	0.0003
	lSMG-lLPFC	3.31	0.0055
	lSMG-rPPC	2.89	0.0122
	rRPFC-rLPFC	3.54	0.0062
	rRPFC-rPPC	2.98	0.0161
	ACC-rPPC	4.74	0.0004
	ACC-rLPFC	4.72	0.0004
	ACC-lLPFC	3.49	0.0050
SN-DMN	rAInsula-rLP	−5.74	0.0000
	rAInsula-lLP	−5.13	0.0001
	lSMG-MPFC	−3.33	0.0055
	lAInsula-lLP	−3.81	0.0030
	lAInsula-rLP	−2.96	0.0169
	rRPFC-rLP	−2.38	0.0479
	lRPFC-PCC	3.51	0.0053
SN-DAN	rAInsula-rIPS	3.23	0.0080
	rSMG-rIPS	4.77	0.0004
	lSMG-lIPS	5.87	0.0000
	lSMG-rIPS	3.12	0.0078
	rRPFC-rIPS	2.43	0.0479
FPN-DMN	lPPC-rLP	9.86	0.0000
	lLPFC-rLP	5.62	0.0001
	rPPC-rLP	5.22	0.0001
	rLPFC-rLP	3.02	0.0129
	PCC-rPPC	3.59	0.0038
	rPPC-lLP	11.68	0.0000
	lPPC-lLP	5.31	0.0001
	lLPFC-lLP	4.80	0.0003
	PCC-lPPC	3.06	0.0119
	lLP-rLPFC	4.65	0.0005
FPN-DAN	lPPC-lIPS	3.31	0.0075
	lLPFC-lIPS	2.65	0.0334
DAN-DMN	rIPS-rLP	−3.98	0.0031
	rIPS-lLP	−2.75	0.0269

*FDR, false discovery rate; ROI, region of interest; RW, rested wakefulness. *p* < 0.05 FDR set wise corrected for all comparisons across the entire network. DAN, dorsal attention network; DMN, default mode network; FPN, fronto-parietal network; SN, salience network.*

**TABLE 4:** Resting state functional connectivity ROI pairs within- and between- DAN, DMN, FPN, and SN network for TSD scans (*t*-test).

**Network**	**ROI-to-ROI**	***t***	**p-FDR**
Within-SN	ACC-lAInsula	11.44	0.0000
	ACC-lRPFC	4.03	0.0018
	ACC-lSMG	3.73	0.0028
	ACC-rAInsula	7.87	0.0000
	ACC-rRPFC	3.83	0.0025
	ACC-rSMG	4.12	0.0017
	lAInsula-lRPFC	6.02	0.0000
	lAInsula−lSMG	7.79	0.0000
	lAInsula-rAInsula	11.61	0.0000
	lAInsula-rRPFC	4.70	0.0003
	lAInsula-rSMG	5.18	0.0001
	lRPFC-lSMG	5.48	0.0001
	lRPFC-rAInsula	3.88	0.0026
	lRPFC-rRPFC	8.92	0.0000
	lRPFC-rSMG	3.39	0.0072
	lSMG-rAInsula	4.30	0.0010
	lSMG-rRPFC	3.42	0.0046
	lSMG-rSMG	8.12	0.0000
	rAInsula-rRPFC	7.72	0.0000
	rAInsula-rSMG	8.66	0.0000
	rRPFC-rSMG	8.07	0.0000
Within-FPN	lLPFC-lPPC	7.51	0.0000
	lLPFC-rLPFC	4.90	0.0003
	lLPFC-rPPC	6.21	0.0000
	lPPC-rLPFC	6.50	0.0000
	lPPC-rPPC	11.36	0.0000
	rLPFC-rPPC	6.30	0.0000
Within-DMN	lLP-MPFC	6.18	0.0000
	lLP-PCC	6.52	0.0000
	lLP-rLP	13.70	0.0000
	MPFC-PCC	5.60	0.0001
	MPFC-rLP	6.86	0.0000
	PCC-rLP	5.35	0.0002
Within-DAN	lFEF-lIPS	4.31	0.0028
	lFEF-rFEF	5.42	0.0004
	lFEF-rIPS	3.03	0.0230
	lIPS-rFEF	4.11	0.0018
	lIPS-rIPS	13.56	0.0000
	rFEF-rIPS	3.69	0.0062
SN-FPN	ACC-lLPFC	4.64	0.0006
	ACC-lPPC	2.60	0.0302
	ACC-rLPFC	4.88	0.0005
	lAInsula-lLPFC	3.80	0.0021
	rSMG-rLPFC	4.11	0.0012
	lRPFC-lLPFC	5.04	0.0002
	lSMG-lPPC	6.43	0.0000
	lSMG-lLPFC	3.27	0.0060
	lSMG-rPPC	2.96	0.0113
	rAInsula-rLPFC	3.41	0.0053
	ACC-rPPC	3.52	0.0041
	rRPFC-rLPFC	5.79	0.0000
	rRPFC-rPPC	2.41	0.0450
	rSMG-rPPC	4.07	0.0012
SN-DMN	lAInsula-lLP	−3.94	0.0017
	lAInsula-PCC	−2.50	0.0311
	lAInsula-rLP	−2.64	0.0248
	lRPFC−PCC	3.03	0.0142
	lSMG-MPFC	−3.53	0.0040
	rAInsula-lLP	−3.32	0.0058
	rAInsula-PCC	−2.58	0.0283
	rAInsula-rLP	−5.32	0.0001
	rRPFC-PCC	2.71	0.0271
	rRPFC-rLP	−2.70	0.0271
SN-DAN	lAInsula-lIPS	4.73	0.0003
	lAInsula-rIPS	3.45	0.0043
	lSMG-lIPS	8.92	0.0000
	lSMG-rIPS	3.95	0.0019
	rAInsula-lIPS	2.29	0.0491
	rAInsula-rIPS	6.61	0.0000
	rSMG-rIPS	7.79	0.0000
	rSMG-lIPS	2.97	0.0132
FPN-DMN	lLPFC-lLP	3.15	0.0086
	rPPC- lLP	10.79	0.0000
	lPPC-lLP	4.63	0.0004
	lPPC-PCC	2.47	0.0445
	rLPFC-PCC	2.83	0.0181
	lLPFC- rLP	5.50	0.0001
	lPPC-rLP	9.55	0.0000
	rPPC-rLP	3.77	0.0034
FPN-DAN	lPPC-lIPS	3.94	0.0019
	lLPFC-lIPS	3.69	0.0035
	rLPFC-lFEF	−3.80	0.0027
	rLPFC-rFEF	−3.03	0.0126
	rPPC-lFEF	−3.63	0.0035
	rPPC-rFEF	−3.68	0.0035

*FDR, false discovery rate; ROI, region of interest; TSD, total sleep deprivation. *p* < 0.05 FDR set wise corrected for all comparisons across the entire network. DAN, dorsal attention network; DMN, default mode network; FPN, fronto-parietal network; SN, salience network.*

### Changes in Four Brain Network Connections Before and After SD

**FIGURE 5:**
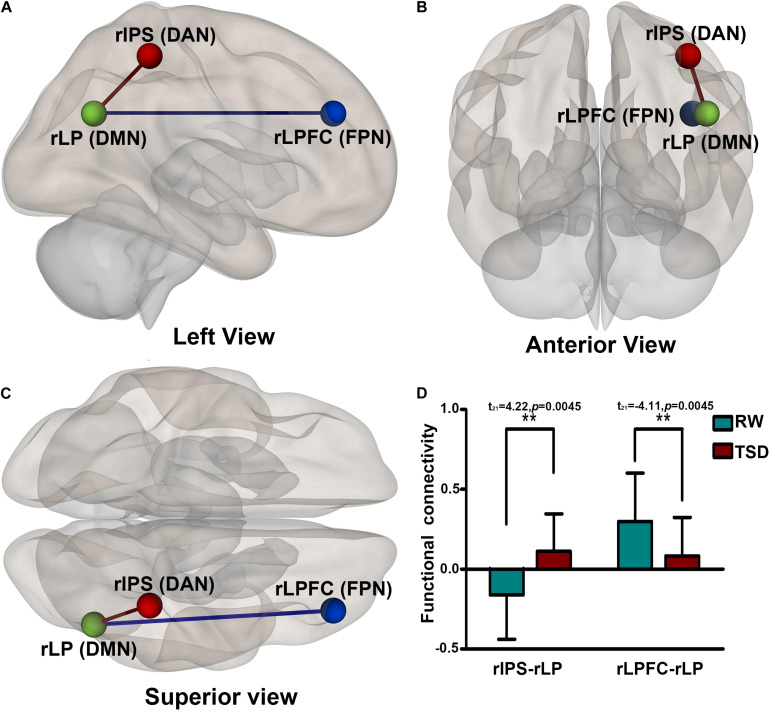
Alerted ROI-to-ROI functional connectivity of networks contrasting in RW vs. TSD scans: Comparing to RW scan, functional connectivity of rIPS-rLP (DAN-DMN) is higher (the red line), and functional connectivity of rLPFC-rLP (FPN-DMN) is lower (the blue line). All the results are shown at panels **(A)** left, **(B)** anterior, **(C)** Superior view, **(D)** shows the effect size of functional connectivity. [**p < 0.05 FDR set wise corrected for all comparisons across the entire network]. ROI, region of interest; TSD, total sleep deprivation; RW, rested wakefulness; rIPS, right intraparietal sulcus; DAN, dorsal attention network; rLP, right paracentral lobule; DMN, default mode network; rLPFC, right lateral prefrontal cortex; FPN, fronto-parietal network.

After TSD, the functional connectivity was higher between DAN and DMN (rIPS-rLP) (*t*_21_ = 4.22, *p* = 0.0045, FDR-corrected) and lower between FPN and DMN (rLPFC-rLP) (*t*_21_ = −4.11, *p* = 0.0045, FDR-corrected) when compared with the RW condition. The results are shown in Figure 5 and Table 5.

**TABLE 5:** ROI-to-ROI functional connectivity statistics of network: comparisons between RW and TSD scans (*t*-test).

**Network**	**ROI-to-ROI**	***t***	**p-FDR**
DAN-DMN	rIPS-rLP	4.22	0.0045
FPN-DMN	rLPFC-rLP	−4.11	0.0045

*ROI, region of interest; FDR, false discovery rate; RW, rested wakefulness; TSD, total sleep deprivation. *p* < 0.005 FDR set wise corrected for all comparisons across the entire network. DAN, dorsal attention network; DMN, default mode network; FPN, fronto-parietal network; SN, salience network.*

### Correlations Between Changes in Strength of Connectivity and WM

**FIGURE 6:**
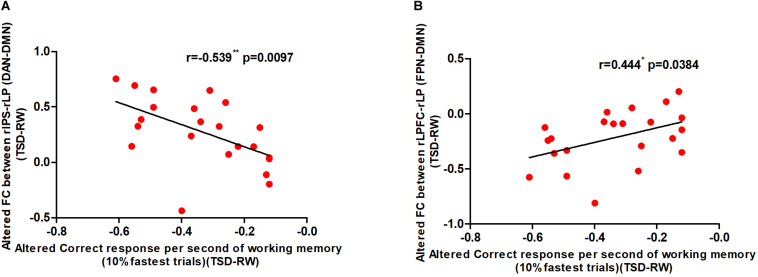
Alerted functional connectivity correlate to altered correct response time of working memory: Alerted correct response per second of working memory is **(A)** negatively correlated with the alerted functional connectivity of rIPS-rLP (DAN-DMN), and **(B)** positively correlated with the alerted functional connectivity of rLPFC-rLP (FPN-DMN) [*p < 0.05 and **p < 0.01]. TSD, total sleep deprivation; RW, rested wakefulness; rIPS, right intraparietal sulcus; DAN, dorsal attention network; rLP, right paracentral lobule; DMN, default mode network; rLPFC, right lateral prefrontal cortex; FPN, fronto-parietal network (10% fastest trials of working memory).

In order to explore the relationship between changes in functional connectivity and WM, we calculated the correlation between the change in correct responses per second of WM and that of the two functional connectivity before and after SD. The change in the number of correct responses per second was negatively correlated with increased functional connectivity of rIPS-rLP (DAN-DMN) and positively correlated with decreased functional connectivity of rLPFC-rLP (FPN-DMN). The results are shown in Figure 6.

## Discussion

Our study analyzed functional connectivity in the resting state of healthy subjects before and after SD. We found that functional connectivity increased between the DAN and DMN after SD and decreased between the FPN and DMN networks. Moreover, there was a significant positive relationship between enhancement of functional connectivity between the DAN and DMN and the decline in WM performance and a significant negative relationship between decreased functional connectivity between the FPN and DMN and the decline in WM performance. This finding indicates that enhanced connectivity between the DAN and DMN may have an important effect on the decline in WM after SD.

The main finding of this study was that SD enhances the functional connectivity between DAN and DMN and that this change is significantly related to a decline in WM performance. From the perspective of the WM model, DAN should be the core brain network for processing and maintaining visuospatial sketchpad (Fairhall et al., 2009; Tamber-Rosenau et al., 2018). The main function of DAN is to provide top-down attention to the outside world and participate in the completion of exogenous tasks (Ptak and Schnider, 2010; Fornito et al., 2012). The main function of DMN is the individual’s continuous attention to self, which antagonizes the function of DAN (Lei et al., 2013). DMN mainly includes the PFC, PCC, and ACC, which is the neural basis of the self and is related to maintaining the awake state (Moran et al., 2013). DMN is very special among brain networks because it is often more activated in the resting state than in the task state (Qin and Northoff, 2011). One possible explanation for this is that the DMN can interfere with attention to specific tasks (Schilbach et al., 2008). The enhanced functional connection between the DAN and DMN found in this study indicates that SD reduces the ability of the individual to distinguish between internal and external sources, which may result in inefficient allocation of attention resources. Moreover, there was a significant negative relationship between the enhanced connectivity between the DAN and DMN and the change in WM scores, confirming that changes in brain network connectivity do affect cognitive function in humans.

Another main finding of this study was that the functional connectivity between FPN and DMN declines after SD, which is in accordance with previous research showing that the functional connectivity of DMN and its anti-networks declines after SD (Gujar et al., 2010; Havas et al., 2012). Further analysis found a significant positive relationship between this decline and changes in WM performance. FPN does not have left-right symmetry; hence, we need to distinguish the right FPN from the left. The left FPN is mainly related to verbal expression and memory (Zhu et al., 2017), and the right FPN basically participates in any task that requires cognitive control, such as WM and activity suppression (Seo et al., 2012). The FPN is considered a central actuator in the WM model and responsible for the macro-control of WM information processing (Sakagami and Tsutsui, 1999; Jones and Harrison, 2001; Wang et al., 2010; Jemery and Volker, 2019). There is a difference between DAN and FPN in terms of WM tasks that require constant monitoring and manipulation of information. In short, DAN is mainly involved in information manipulation, while FPN plays a more important role in information monitoring (Champod and Petrides, 2010).

Our results are in line with those of many other behavioral studies (Van et al., 2012; Lu, 2016; Tempesta et al., 2017; Harrington et al., 2018) but not all of them. Dunsmoor et al. (2015) found that sleep could selectively effect memory of related events but that sleep is not necessary to strengthen memory. The time interval between the experience itself and recall may be more important. Gerhardsson et al. (2019) found no impairment of WM in sleep-deprived elderly subjects but found differences in effects of SD on WM in people of different ages. In an electroencephalographic (EEG) study, memory performance worsened after SD and there was a significant change in the correlation between the left and right PFC, indicating that SD impairs memory by affecting the PFC (Li et al., 2014). This result is consistent with the finding by Yeung et al. (2018) in a study using near-infrared spectroscopy (NIRS) that the frontal cortex was inhibited after SD. In studies of event-related potentials, it was found that performance on memory tasks decreased after SD and that there was a change in the values of some EEG components, including N_2_, P_3_, and P_6_ (Mograss et al., 2008, 2009; Qi et al., 2010).

Neuroimaging studies of SD have found that many changes in cognitive function are related to changes in DMN and other brain network connections (Havas et al., 2012; Esposito et al., 2018; Jemery and Volker, 2019). Our research found similar results. WM models address situations where cognitive resources are sufficient and cognitive function is adjusted. However, it has been found that these models are affected by significant changes in attention resources after SD (Anderson and Horne, 2006; Lim and Dinges, 2010). Therefore, attentional resource factors should be added to this model to make it applicable to interpretation of generation of WM and changes in its mechanisms under a wider range of conditions. Future research should evaluate the correlation between collaborative changes in brain networks and changes in WM under different attention resources.

## Limitations

The main shortcoming of this study is that it only recruited young male subjects, so its results cannot be extrapolated to women or individuals in other age groups. Previous studies have shown that effects of SD on WM can vary with age (Gerhardsson et al., 2019). Therefore, subsequent research should include subjects of both sexes and a broader range of ages. Second, although we asked the subjects to stay awake during the resting scan and took measures to keep them awake, we cannot exclude the possibility that some may have fallen asleep during the scanning procedure. If this occurred, the effect of SD will be affected. Therefore, future researchers should include EEG to obtain objective data to identify whether the subjects actually fall asleep. Third, we used only the 2-back paradigm to measure WM, which cannot analyze changes in WM load and functional connectivity within the brain network.

## Conclusion

In short, current study observed significant changes in connections between brain networks and found a link between changes in brain networks and changes in WM after SD. This may help us to clarify the brain network mechanism that sleep affects WM, the relationship between different brain regions and brain networks and WM, and to treat some memory disorders.

## Data Availability Statement

The datasets generated for this study are available on request to the corresponding author.

## Ethics Statement

The studies involving human participants were reviewed and approved by the Ethics Committee of The General Hospital of PLA (Beijing, China). The patients/participants provided their written informed consent to participate in this study.

## Author Contributions

CD and YZ conceptualized, investigated, visualized the data, carried out the formal analysis, and wrote the manuscript. XC, ZP, and LZ conceptualized and investigated the data. YS conceptualized and supervised the data and carried out the funding acquisition and project administration. CW conceptualized the data and carried out the funding acquisition and project administration. All authors contributed to the article and approved the submitted version.

## Conflict of Interest

The authors declare that the research was conducted in the absence of any commercial or financial relationships that could be construed as a potential conflict of interest.

## Footnotes

1 http://www.fil.ion.ucl.ac.uk/spm/

2 https://www.nitrc.org/projects/conn

## References

[B1] Anderson C. Horne J. A. (2006). Sleepiness enhances distraction during a monotonous task. *Sleep* 29 573–576. 10.1093/sleep/29.4.573 16676792

[B2] Baddeley A. (2007). *Oxford Psychology Series: Vol. 45. Working Memory, Thought, and Action.* Oxford: Oxford University Press, 10.1093/acprof:oso/9780198528012.001.0001

[B3] Baddeley A. (2010). Working memory. *Curr. Biol.* 20 R136–R140. 10.1016/j.cub.2009.12.014 20178752

[B4] Baddeley A. (2012). Working memory: theories, models, and controversies. *Annu. Rev. Psychol.* 63 1–29. 10.1146/annurev-psych-120710-100422 21961947

[B5] Badre D. Wagner A. D. (2007). Left ventrolateral prefrontal cortex and the cognitive control of memory. *Neuropsychologia* 45 2883–2901. 10.1016/j.neuropsychologia.2007.06.015 17675110

[B6] Basner M. Dinges D. F. (2011). Maximizing sensitivity of the psychomotor vigilance test (PVT) to sleep loss. *Sleep* 34 581–591. 10.1093/sleep/34.5.581 21532951 PMC3079937

[B7] Behzadi Y. Restom K. Liau J. Liu T. T. (2007). A component based noise correction method (compcor) for bold and perfusion based fmri. *Neuroimage* 37 90–101. 10.1016/j.neuroimage.2007.04.042 17560126 PMC2214855

[B8] Biswal B. B. (2012). Resting state fMRI: a personal history. *Neuroimage* 62 938–944. 10.1016/j.neuroimage.2012.01.090 22326802 PMC12911935

[B9] Blumenfeld R. Ranganath C. (2007). Prefrontal cortex and long-term memory encoding: an integrative review of findings from neuropsychology and neuroimaging. *Neurosci. Rev. J. Bring. Neurobiol. Neurol. Psychiatr.* 13:280. 10.1177/1073858407299290 17519370

[B10] Boardman J. M. Bei B. Mellor A. Anderson C. Sletten T. L. Drummond S. P. A. (2018). The ability to self-monitor cognitive performance during 60 h total sleep deprivation and following 2 nights recovery sleep. *J. Sleep Res.* 27:e12633. 10.1111/jsr.12633 29159907

[B11] Bunge S. A. Carter W. David B. Wagner A. D. (2005). Analogical reasoning and prefrontal cortex: evidence for separable retrieval and integration mechanisms. *Cereb. Cortex* 15 239–249. 10.1093/cercor/bhh126 15238433

[B12] Cappell K. A. Gmeindl L. Reuter-Lorenz P. A. (2010). Age differences in prefrontal recruitment during verbal working memory maintenance depend on memory load. *Cortex* 46:473. 10.1016/j.cortex.2009.11.009 20097332 PMC2853232

[B13] Champod A. S. Petrides M. (2010). Dissociation within the frontoparietal network in verbal working memory: a parametric functional magnetic resonance imaging study. *J. Neurosci.* 30 3849–3856. 10.1523/JNEUROSCI.0097-10.2010 20220020 PMC6632229

[B14] Chee M. W. L. Chuah L. Y. M. Venkatraman V. Chan W. Y. Philip P. Dinges D. F. (2006). Functional imaging of working memory following normal sleep and after 24 and 35 h of sleep deprivation: correlations of fronto-parietal activation with performance. *Neuroimage* 31 419–428. 10.1016/j.neuroimage.2005.12.001 16427321

[B15] Chein J. M. Ravizza S. M. Fiez J. A. (2003). Using neuroimaging to evaluate models of working memory and their implications for language processing. *J. Neurolinguist.* 16 315–339. 10.1016/S0911-6044(03)00021-6

[B16] Chen W. H. Chen J. Lin X. Li P. Shi L. Shi J. (2018). Dissociable effects of sleep deprivation on functional connectivity in the dorsal and ventral default mode networks. *Sleep Med.* 50 137–144. 10.1016/j.sleep.2018.05.040 30055480

[B17] Collette F. Linden M. V. D. (2002). Brain imaging of the central executive component of working memory. *Neurosci. Biobehav. Rev.* 26 105–125. 10.1016/S0149-7634(01)00063-X 11856556

[B18] Costers L. Van Schependom J. Laton J. Baijot J. Sjøgård M. Wens V. (2020). Spatiotemporal and spectral dynamics of multi-item working memory as revealed by the n-back task using MEG. *Hum. Brain Mapp.* 41 2431–2446. 10.1002/hbm.24955 32180307 PMC7267970

[B19] Cowan N. (1999). “An embedded-processes model of working memory,” in *Models of Working Memory: Mechanisms of Active Maintenance and Executive Control*, eds Miyake A. Shah P. (Cambridge: Cambridge University Press), 62–101. 10.1017/CBO9781139174909.006

[B20] Curtis C. E. D’esposito M. (2003). Persistent activity in the prefrontal cortex during working memory. *Trends Cogn. Sci.* 7 415–423. 10.1016/S1364-6613(03)00197-9 12963473

[B21] Drummond S. P. A. Anderson D. E. Straus L. D. Vogel E. K. Perez V. B. (2012). The effects of two types of sleep deprivation on visual working memory capacity and filtering efficiency. *PLoS One* 7:e35653. 10.1371/journal.pone.0035653 22530064 PMC3329471

[B22] Dunsmoor J. E. Murty V. P. Davachi L. Phelps E. A. (2015). Emotional learning selectively and retroactively strengthens memories for related events. *Nature* 520 345–348. 10.1038/nature14106 25607357 PMC4432479

[B23] Esposito R. Cieri F. Chiacchiaretta P. Cera N. Lauriola M. Di Giannantonio M. (2018). Modifications in resting state functional anticorrelation between default mode network and dorsal attention network: comparison among young adults, healthy elders and mild cognitive impairment patients. *Brain Imag. Behav.* 12 127–141. 10.1007/s11682-017-9686-y 28176262

[B24] Fairhall S. L. Indovina I. Driver J. Macaluso E. (2009). The brain network underlying serial visual search: comparing overt and covert spatial orienting, for activations and for effective connectivity. *Cereb. Cortex* 19 2946–2958. 10.1093/cercor/bhp064 19395524

[B25] Fornito A. Harrison B. J. Zalesky A. Simons J. S. (2012). Competitive and cooperative dynamics of large-scale brain functional networks supporting recollection. *Proc. Natl. Acad. Sci. U.S.A.* 109 12788–12793. 10.1073/pnas.1204185109 22807481 PMC3412011

[B26] Frenda S. J. Patihis L. Loftus E. F. Lewis H. C. Fenn K. M. (2014). Sleep deprivation and false memories. *Psychology* 25 1674–1681. 10.1177/0956797614534694 25031301

[B27] Gerhardsson A. Fischer H. Lekander M. Kecklund G. Axelsson J. Åkerstedt T. (2019). Positivity effect and working memory performance remains intact in older adults after sleep deprivation. *Front. Psychol.* 10:605. 10.3389/fpsyg.2019.00605 30967813 PMC6440387

[B28] Greicius M. D. Krasnow B. Reiss A. L. Menon V. (2003). Functional connectivity in the resting brain: a network analysis of the default mode hypothesis. *Proc. Natl. Acad. Sci. U.S.A.* 100 253–258. 10.1073/pnas.0135058100 12506194 PMC140943

[B29] Gujar N. Yoo S. S. Hu P. Walker M. P. (2010). The unrested resting brain: sleep deprivation alters activity within the default-mode network. *J. Cogn. Neurosci.* 22 1637–1648. 10.1162/jocn.2009.21331 19702469 PMC2883887

[B30] Harrington M. O. Nedberge K. M. Durrant S. J. (2018). The effect of sleep deprivation on emotional memory consolidation in participants reporting depressive symptoms. *Neurobiol. Learn. Mem.* 152:13. 10.1016/j.nlm.2018.04.013 29709569

[B31] Havas J. A. D. Parimal S. Soon C. S. Chee M. W. L. (2012). Sleep deprivation reduces default mode network connectivity and anti-correlation during rest and task performance. *Neuroimage* 59 1745–1751. 10.1016/j.neuroimage.2011.08.026 21872664

[B32] Heuvel M. P. V. D. Pol H. E. H. (2010). Exploring the brain network: a review on resting-state fMRI functional connectivity. *Eur. Neuropsychopharmacol.* 20 519–534. 10.1016/j.euroneuro.2010.03.008 20471808

[B33] Jansma J. M. Ramsey N. F. Coppola R. René S. K. (2000). Specific versus nonspecific brain activity in a parametric n-back task. *Neuroimage* 12 688–697. 10.1006/nimg.2000.0645 11112400

[B34] Jemery M. T. Volker N. (2019). Cortico-limbic pain mechanisms. *Neurosci. Lett.* 702 15–23. 10.1016/jneulet.2018.11.037 30503916 PMC6520155

[B35] Jones K. Harrison Y. (2001). Frontal lobe function, sleep loss and fragmented sleep. *Sleep Med. Rev.* 5:475. 10.1053/smrv.2001.0203 12531154

[B36] Kusztor A. Raud L. Juel B. E. Nilsen A. S. Storm J. F. Huster R. J. (2019). Sleep deprivation differentially affects subcomponents of cognitive control. *Sleep* 42:zsz016. 10.1093/sleep/zsz016 30649563

[B37] Lei X. Zhao Z. Chen H. (2013). Extraversion is encoded by scale-free dynamics of default mode network. *Neuroimage* 74 52–57. 10.1016/j.neuroimage.2013.02.020 23454049

[B38] Li M. Gao Y. Gao F. Anderson A. W. Ding Z. Gore J. C. (2020). Functional engagement of white matter in resting-state brain networks. *Neuroimage* 220:117096. 10.1016/j.neuroimage.2020.117096 32599266 PMC7594260

[B39] Li N. Zhang J. M. Liu X. Y. (2014). Research of EEG features induced by the effects of sleep deprivation on vision short-term memory. *Modern Instrum. Med. Treat.* 20 5–8. 10.11876/mimt201401002

[B40] Lim J. Dinges D. F. (2010). A meta-analysis of the impact of short-term sleep deprivation on cognitive variables. *Psychol. Bull.* 136 375–389. 10.1037/a0018883 20438143 PMC3290659

[B41] Lu F. M. Cui Q. Huang X. J. Li L. Y. Duan X. J. Chen H. F. (2020). Anomalous intrinsic connectivity within and between visual and auditory networks in major depressive disorder. *Prog. Neuro Psychopharmacol. Biol. Psychiatry* 100:109889. 10.1016/j.pnpbp.2020.109889 32067960

[B42] Lu L. (2016). The effect of 72 h of sleep deprivation on the iowa gambling task. *Noropsikiyatri Arsivi* 53:357. 10.5152/npa.2016.12505 28360813 PMC5353045

[B43] Luerding R. Weigand T. Bogdahn U. Schmidt-Wilcke T. (2008). Working memory performance is correlated with local brain morphology in the medial frontal and anterior cingulate cortex in fibromyalgia patients: structural correlates of pain-cognition interaction. *Brain* 131(Pt 12), 3222–3231. 10.1093/brain/awn229 18819988

[B44] Martínez-Cancino D. P. Azpiroz-Leehan J. Jiménez-Angeles L. (2015). The effects of sleep deprivation in working memory using the n-back task. *IFMBE Proc.* 49 421–424. 10.1007/978-3-319-13117-7_108

[B45] Mograss M. A. Guillem F. Brazzini-Poisson V. Godbout R. (2009). The effects of total sleep deprivation on recognition memory processes: a study of event-related potential. *Neurobiol. Learn. Mem.* 91 343–352. 10.1016/j.nlm.2009.01.008 19340944

[B46] Mograss M. A. Guillem F. Godbout R. (2008). Event-related potentials differentiates the processes involved in the effects of sleep on recognition memory. *Psychophysiology* 45 420–434. 10.1111/j.1469-8986.2007.00643.x 18221442

[B47] Moran J. M. Kelley W. M. Heatherton T. F. (2013). What can the organization of the brain’s default mode network tell us about self-knowledge? *Front. Hum. Neurosci.* 7:391. 10.3389/fnhum.2013.00391 23882210 PMC3713343

[B48] Mu Q. Nahas Z. Johnson K. A. Yamanaka K. Mishory A. Koola J. (2005). Decreased cortical response to verbal working memory following sleep deprivation. *Sleep* 28 55–67. 10.1093/sleep/28.1.55 15700721

[B49] Oberauer K. (2002). Access to information in working memory: exploring the focus of attention. *J. Exper. Psychol. Learn. Mem. Cogn.* 28 411–421. 10.1037/0278-7393.28.3.411 12018494

[B50] Owen A. M. McMillan K. M. Laird A. R. Bullmore E. (2005). N-back working memory paradigm: a meta-analysis of normative functional neuroimaging studies. *Hum. Brain Mapp.* 25 46–59. 10.1002/hbm.20131 15846822 PMC6871745

[B51] Preece D. (2012). The effect of working memory (n-back) training on fluid intelligence. *Front. Hum. Neurosci.* 6 81–89. 10.3389/fnhum.2012.00166 22514529 PMC3324108

[B52] Ptak R. Schnider A. (2010). The dorsal attention network mediates orienting toward behaviorally relevant stimuli in spatial neglect. *J. Neurosci.* 30 12557–12565. 10.1523/JNEUROSCI.2722-10.2010 20861361 PMC6633576

[B53] Qi J. L. Shao Y. C. Miao D. Fan M. Bi G. H. Yang Z. (2010). The effects of 43 hours of sleep deprivation on executive control functions: event-related potentials in a visual go/no go task. *Soc. Behav. Person. Intern. J.* 38 29–42. 10.2224/sbp.2010.38.1.29

[B54] Qin P. Northoff G. (2011). How is our self related to midline regions and the default-mode network? *Neuroimage* 57 1221–1233. 10.1016/j.neuroimage.2011.05.028 21609772

[B55] Roberts B. M. Libby L. A. Inhoff M. C. Ranganath C. (2017). Brain activity related to working memory for temporal order and object information. *Behav. Brain Res.* 354 55–63. 10.1016/j.bbr.2017.05.068 28602963

[B56] Sakagami M. Tsutsui K. (1999). The hierarchical organization of decision making in the primate prefrontal cortex. *Neurosci. Res.* 34:79 10.1016/S0168-0102(99)00038-3 10498334

[B57] Schilbach L. Eickhoff S. B. Rotarska-Jagiela A. Fink G. R. Vogeley K. (2008). Minds at rest? social cognition as the default mode of cognizing and its putative relationship to the “default system” of the brain. *Conscious. Cogn.* 17 457–467. 10.1016/j.concog.2008.03.013 18434197

[B58] Seidman L. J. Thermenos H. W. Poldrack R. A. Peace N. K. Koch J. K. Tsuang M. T. (2006). Altered brain activation in dorsolateral prefrontal cortex in adolescents and young adults at genetic risk for schizophrenia: an fmri study of working memory. *Schizophr. Res.* 85 58–72. 10.1016/j.schres.2006.03.019 16632333

[B59] Seo J. Kim S. H. Kim Y. T. Song H. J. Lee J. J. Kim S. H. (2012). Working memory impairment in fibromyalgia patients associated with altered frontoparietal memory network. *PLoS One* 7:e37808. 10.1371/journal.pone.0037808 22715371 PMC3370998

[B60] Soveri A. Karlsson E. P. A. Waris O. Grönholm-Nyman P. Laine M. (2017). Pattern of near transfer effects following working memory training with a dual n-back task. *Exper. Psychol.* 64 240–252. 10.1027/1618-3169/a000370 28922999

[B61] Tamber-Rosenau B. J. Asplund C. L. Rene M. (2018). Functional dissociation of the inferior frontal junction from the dorsal attention network in top-down attentional control. *J. Neurophysiol.* 120 2498–2512. 10.1152/jn.00506.2018 30156458 PMC6295539

[B62] Tempesta D. Socci V. Dello Ioio G. De Gennaro L. Ferrara M. (2017). The effect of sleep deprivation on retrieval of emotional memory: a behavioural study using film stimuli. *Exper. Brain Res.* 235 3059–3067. 10.1007/s00221-017-5043-z 28741085

[B63] Terán-Pérez G. J. Ruiz-Contreras A. E. González-Robles R. O. Tarrago-Castellanos R. Mercadillo R. E. Jiménez-Anguiano A. (2012). Sleep deprivation affects working memory in low but not in high complexity for the N-Back test. *Neurosci. Med.* 03 380–386. 10.4236/nm.2012.34047

[B64] Van D. E. V. Jan-Willem T. Atsuko T. Markus B. Fernández Guillén L. Kim F. (2012). Sleep supports selective retention of associative memories based on relevance for future utilization. *PLoS One* 7:e43426. 10.1371/journal.pone.0043426 22916259 PMC3420871

[B65] Wang L. Liu X. Guise K. G. Knight R. T. Ghajar J. Fan J. (2010). Effective connectivity of the fronto-parietal network during attentional control. *J. Cogn. Neurosci.* 22 543–553. 10.1162/jocn.2009.21210 19301995

[B66] Whitfield-Gabrieli S. Nieto-Castanon A. (2012). Conn: a functional connectivity toolbox for correlated and anticorrelated brain networks. *Brain Connect.* 2 125–141. 10.1089/brain.2012.0073 22642651

[B67] Wu G. Wang Y. Mwansisya T. E. Pu W. Zhang H. (2014). Effective connectivity of the posterior cingulate and medial prefrontal cortices relates to working memory impairment in schizophrenic and bipolar patients. *Schizophr. Res.* 158 85–90. 10.1016/j.schres.2014.06.033 25043264

[B68] Yeung M. K. Lee T. L. Cheung W. K. Chan A. S. (2018). Frontal underactivation during working memory processing in adults with acute partial sleep deprivation: a near-infrared spectroscopy study. *Front. Psychol.* 9:742. 10.3389/fpsyg.2018.00742 29867694 PMC5964163

[B69] Yoon J. H. Grandelis A. Maddock R. J. (2016). Dorsolateral prefrontal cortex gaba concentration in humans predicts working memory load processing capacity. *J. Neurosci.* 36 11788–11794. 10.1523/JNEUROSCI.1970-16.2016 27852785 PMC5125231

[B70] Zhu W. Chen Q. Xia L. Beaty R. E. Yang W. Tian F. (2017). Common and distinct brain networks underlying verbal and visual creativity. *Hum. Brain Mapp.* 38 2094–2111. 10.1002/hbm.23507 28084656 PMC6866727

